# Secular Trends in Growth of Preschool Children from Rural Maharashtra, India

**DOI:** 10.3329/jhpn.v30i4.13325

**Published:** 2012-12

**Authors:** Shobha Rao, Asawari N. Kanade, Smita B. Joshi, Jayshree S. Sarode

**Affiliations:** Biometry and Nutrition Unit, Agharkar Research Institute, Pune 411004, India

**Keywords:** Height, Preschool children, Secular growth change, Weight, India

## Abstract

The study examined the secular trends in growth of preschool children from rural Maharashtra, India, during 1985-2001. Anthropometric data collected on preschool (<6 years old) children during 2001 (n=1,171) and 1985 (n=979) from the same villages were compared. Decadal change increased with age and was marginally higher in boys than girls. It was the lowest among infants (-0.1 to 0.1 kg and 0.4 to 0.7 cm in both sexes) and the highest among boys of 4+ years (1.3 kg and 2.9 cm) and girls of 5+ years (1.2 kg and 2.1 cm). Increase in weight was higher (10-15%) compared to that in height (3-5%) and, consequently, reduction in the prevalence of wasting was marked (around 68% in boys and 48% in girls) than that in stunting (42% in boys and 27% in girls) among these children. The improvement was higher in boys than in girls. Negligible secular changes in younger children indicate the need for creating health and nutritional awareness among rural mothers while relatively higher improvement in weight than height among older children warns the future possibility of childhood adiposity even among rural populations.

## INTRODUCTION

Positive secular trends in body dimensions and growth rate have been apparent all over the world in both genders during the last century (1). The extent of secular changes has, however, varied during different periods in different populations and in intensity. Most important factors to influence secular growth changes are improvements in environment, food availability, and elimination of chronic hunger. In the 1980s’, secular change has come close to a halt in Bulgaria, Italy, Sweden, Austria, and many developed countries while it continued in Belgium, Czech Republic, Germany, Hungary, and Poland (2). Studies in India investigating secular changes are scarce and more so with preschool children. Most studies have examined such changes for adolescent growth (3), or for menarcheal age in adolescent girls (4) or for the final sizes at adulthood (5,6).

Physical growth in children is one of the best indicators of overall health and well-being of a population. Secular changes in child growth are of primary importance in determining the impact of general social and economic changes and specific public-health interventions. Preschool age being a period for rapid growth would be ideal for observing secular changes. Moreover, it is a sensitive period as growth retardation during early period of life is known to affect the growth significantly during adolescence and the final size at adulthood (7).

India has progressed on several fronts and is believed to pass through a nutritional transition too (8). For example, government data **(**Table 1**)** reveal the changes in macro and micro-level indicators during the 20 years period from 1981 to 2001. First, literacy in the state of Maharashtra increased from 46.7% to 70.84%. In particular, female illiteracy, which is known to be associated with child health and mortality (9), has dropped from 79.34% to 45.84% in rural Maharashtra. In fact, the percentage of young mothers who had completed mid-level school education in our study during 2001 was very high (75%). Second, the developmental changes are notable in terms of the length of *pucca* roads, which has almost doubled during the said period of 15 years. Thus, roads for access to all study villages were improved greatly. Similarly, proportion of houses with electricity has increased by three times. Major occupation in 1981 was agriculture (cultivation and labour selling); 83% of families were engaged in it. However, this proportion reduced in the last 20 years, indicating that almost 35% heads of families in 2001 were engaged in occupations other than agriculture; especially individuals of younger generation were keen in searching jobs in nearby cities. The official data, thus, show a substantial increase from Rs. 2,435 to Rs. 29,204 in per-capita annual income of rural families. Consequently, changes in lifestyle and possession of various assets, like television (24.7%), two/four-wheelers (9.6%), and telephone (4.4%), were notable compared to negligible proportions in 1981.

**Table 1.** Indicators of change in rural Maharashtra during 1981-2001

No.	Indicator	1981	2001
1	Literacy rate (%)^a^	46.70	70.84
	Female literacy rate (Rural India) (%)	20.66^c^	54.16^b^
2	Length of *pucca* roads (km)	141,131^b^	260,000^a^
3	Houses with electricity (%)^a^	24.1	65.2
4	Occupation (%)^a^		
	Agricultural (Cultivation/selling labour)	83.38	16.62
	Other	65.07	34.93
5	Per-capita annual income (Rs.)^d^	2,435	29,204
6	Economic assets (%)^a^		
	TV	-	24.7
	Telephone	-	4.4
	Vehicle (2/3/4-wheeler)	-	9.6
7	Condition of houses (%)^a^		
	Roof (*kaccha*)	73.5	58.6
	Wall (*kaccha*)	66.7	55.3
	Floor (*kaccha*)	94.6	78.6
8	No. of dwelling rooms (%)^a^		
	Less than 2 rooms	65.5	53.6
	≥2 rooms	34.5	46.4
9	Access to safe drinking-water (%)^a^	18.3	68.4
10	Sanitation coverage (Closed drainage and latrine) (%)^e^	1.0	22.0
11	Nutritional status (%)^f^		
	Stunted (0-3)	50.8 (1992)	33.3
	Wasted (0-3)	21.5 (1992)	21.2
12	IMR (per 1,000 livebirths)^g^	69.0 (1991)	55.0

^a^Census of India 2001, Series 28, Maharashtra

^b^1981-2001 Road Development Plan, Road Development in Maharashtra, Public Works Department, Government of Maharashtra

^c^Statistical Database for Literacy – Vol. 2, 1993, National Institute of Adult Education

^d^National Sample Survey for the years 1983, 1993-1994, and 1999-2000

^e^India's national sanitation and hygiene programme: From experience to policy, West Bengal and Maharashtra models provide keys to success, Sumita C Ganguly

^f^Family Health Survey for Maharashtra, IIPS Mumbai, 1995

^g^Economic Survey of Maharashtra, 2004-2005

At the micro-level too, several improvements were seen. For example, type of house improved considerably, and more so with regard to the reduction in houses having *kaccha* roof (73.5% to 58.6%). Similarly, number of houses with more than two dwelling rooms has also increased in the last 20 years. More importantly, significant increase (18.3% to 68.4%) in proportion of houses having access to safe drinking-water was observed. This was not true with regard to sanitation coverage as only 22% of households in 2001 had these facilities. Therefore, despite visible materialistic developments, living conditions in rural areas continue to remain unhygienic. As a result, most health problems in children, viz. childhood malnutrition, especially stunting (33.3%) and infant mortality (55 per 1,000 livebirths) remain to be the major public-health concerns in rural Maharashtra. In the light of the above developments, the study aimed to examine the secular changes in growth of preschool children during 1985 and 2001.

## MATERIALS AND METHODS

### Past study

This study undertaken in 1985 aimed to assess growth of preschool children in rural areas. It covered all children below 6 years of age from seven villages situated within 30 to 40 km from Pune city. In total, 1,171 children were observed for anthropometry and socioeconomic information. Assessment of age was done carefully as most of them needed help to recall the exact month of the child's birth. This was often facilitated by preparing a local calendar based on important festivals or events falling in each month. Children were followed up for anthropometry with a gap of one year (±15 days) for studying the velocity of growth.

All children were measured for weight and height. Body-weight was measured using a portable balance with the accuracy of 50 g while height was measured with 0.1 cm precision with stadiometer. Supine lengths of children below 2 years of age were recorded. For most of the time, the investigators recording a particular measurement were kept same for the entire study period to reduce personal errors.

### Present study

The present study was carried out during 2001 on preschool children from the same villages where the previous study was undertaken. Since estimates of annual increments were available in the previous study, children in the present study were also examined after one year (±15 days) to get estimates of secular changes for growth velocities. In this study, all children (n=979) up to 6 years of age, from the same villages, were measured for weight and height. Body-weight was measured up to 20 g, using digital balance, and height was measured up to 0.1 cm, using stadiometer. Field staff was trained, and inter-observer difference was assessed before starting the main study. Inter as well as intra-observer variation was found to be small (CV<1%). Unlike in the previous study, assessment of age was done on the basis of the cards, giving details of the date and time of birth, issued by the hospital after delivery.

### Statistical analysis

Simple estimates of secular changes were obtained by comparing yearly mean values of weight and height by age and sex-groups in the present and previous study. Different percentile values for the distributions of weight and height in the two studies were computed, and a weighted estimate of secular change was obtained. New growth standards (10) were used in computing weight-for-age and height-for-age z-scores, and mean values were compared using *t*-test. Prevalence of undernutrition and stunting at two time-points was compared using *z*-test. Sample-sizes covered in the previous and the present study are given in Table 2.

**Table 2.** Number of preschool children in the present and the past study

Age (years)	1985	2001
0-1	281	129
>1-3	386	300
>3-5	353	359
≥5	151	191
Total	1,171	979

## RESULTS

**Fig. 1.**
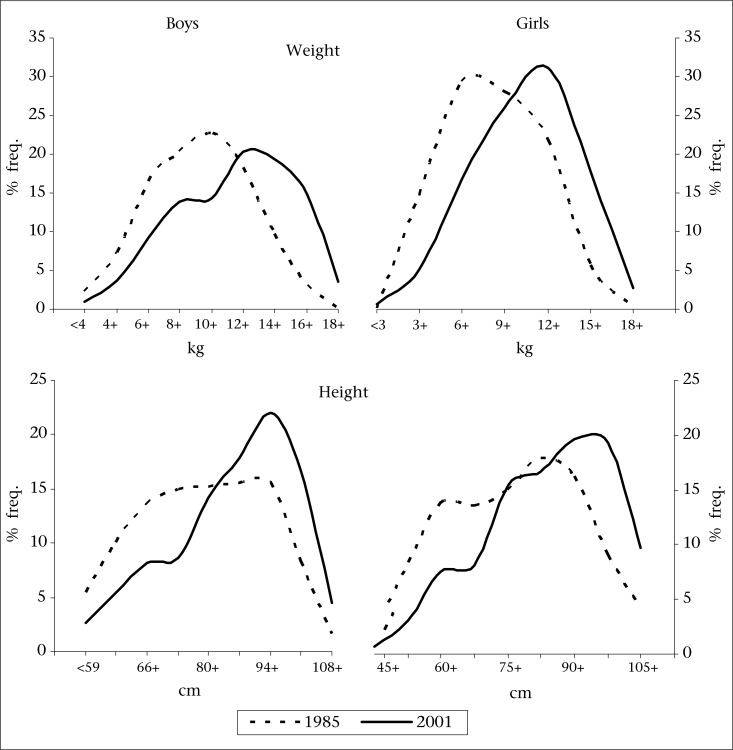
Weight and height distribution among preschool children in two studies—1985 and 2001

**Fig. 2.**
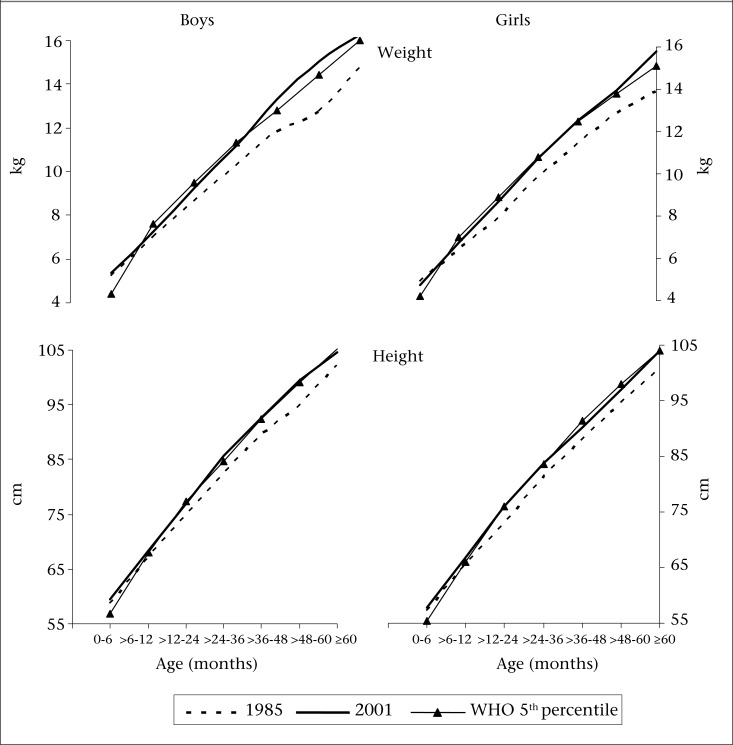
Growth curves for weight and height among preschool children from two studies—1985 and 2001

Distributions of weight and height of children, recorded in the past study and in the present study, are shown for both the sexes in Figure 1. Distributions of weight and height in 2001 showed a shift to the right, indicating the presence of secular changes in these measurements in rural children. The shift in the median of the distribution of weight and height was seen in both the sexes. Mean values for weight and height for respective age-groups observed in the two studies are shown in Figure 2**.** Mean weights for children in 2001 were higher compared to those observed in 1985 for all ages in both the sexes. However, the differences were significant (p<0.01) only beyond infancy for both weight and height in both the sexes (Table 3). The overall growth curves for children in 2001 are comparable to only 5^th^ percentile of WHO (2006) standard.

Estimates of secular changes (decadal changes) in different age-groups were obtained. Age-wise estimates of decadal changes were the lowest for infants both for weight (0.1 kg for boys and −0.11 kg for girls) as well as height (0.4 to 0.7 cm for both the sexes) but increased with age. Thus, in 1 to 3 year(s) of age, the decadal change was around 0.4 to 0.6 kg in both the sexes and increased beyond 3 years to about 1.2 to 1.3 kg in both the sexes. In the case of height also, the decadal change during 1 to 3 year(s) of age was between 1.3 cm to 2.0 cm in both the sexes while beyond 3 years, it increased to 2.9 cm in boys and 2.1 cm in girls. The secular changes in weight and height, thus, increased with age and were marginally higher in boys than girls.

Comparison of values of different percentiles for weight and height distributions observed at two time-points is presented in Fig. 3a and 3b respectively. Since secular trends up to the first two years of age were observed to be smaller than those observed for older children, the comparison is done separately for these two age-groups. It can be observed that, for both the sexes, all the percentile values for weight and height distribution are higher in 2001 study compared to those in 1985 study. Among younger children (<24 months of age), the differences in the 10^th^ percentile values for weight were negligible (0.0 kg and 0.4 kg for boys and girls respectively) and increased beyond the 50^th^ percentile. The difference in the 90^th^ percentile values was 0.8 kg for boys and 1.4 kg for girls. In the case of height, the difference in the 10^th^ percentile values were 1.6 cm and 2.5 cm for boys and girls respectively and increased considerably in the 90^th^ percentile (3.1 cm and 5.6 cm for boys and girls respectively). Thus, in the younger group (<24 months of age), secular changes in height were greater than that in weight and were mainly observed for children in higher percentiles. In contrast, among older children (>24 months of age), the differences in the percentile values were fairly constant across all the percentiles of weight and height distributions.

Mean weight and height-for-age z-scores for (using the 50^th^ percentile of WHO, 2006) children in each age-group improved in 2001 compared to those in 1985 for both the sexes (Fig. 4). However, weight-for-age z-scores rapidly declined from early infancy, were maximum at around 12 months of age and stabilized but did not improve in 1985 in both the sexes and improved significantly, especially among boys than in girls in 2001. In contrast, height-for-age z-scores declined rapidly from early infancy till 30 months of age and remained stable, with little improvement thereafter in both the sexes in 1985 as well as in 2001. Data from both the studies, thus, show a decreasing trend in height-for-age z-scores till the 4^th^ year of age, indicating progression of stunting as age increases.

The maximum negative value of mean weight-for-age z-score was observed at around 12 months while that for height was between 24 and 36 months of age in both the sexes in the present and the past study. Thus, weight-for-age z-scores at 5 years of age improved from −1.82 (in 1985) to −0.91 (in 2001) for boys and −1.56 to −1.14 for girls. Mean height-for-age z scores at the age of five years improved from −2.66 (in 1985) to −1.57 (in 2001) in case of boys and from −2.44 to −1.9 in case of girls over the period of 15 years.

**Table 3.** Differences in weight and height for preschool children by age and sex

Age (months)	Weight (kg)							
	Boys				Girls			
	1985	2001	Difference	Decadal change	1985	2001	Difference	Decadal change
0-6	5.21	5.38	0.17	0.1	4.90	4.73	-0.17	-0.1
>6-12	7.02	7.27	0.25	0.2	6.42	6.76	0.34	0.2
>12-24	8.62	9.26	0.64**	0.4	7.91	8.66	0.75**	0.5
>24-36	10.27	11.18	0.91**	0.5	9.83	10.76	0.93**	0.6
>36-48	11.83	13.30	1.47**	0.9	11.4	12.51	1.11**	0.7
>48-60	12.77	15.03	2.26**	1.3	12.85	13.93	1.08**	0.6
>60-72	14.73	16.23	1.50**	0.9	13.92	15.77	1.85**	1.2
Height (cm)								
0-6	58.5	59.5	0.90	0.5	57.1	57.8	0.70	0.4
>6-12	67.2	68.4	1.20	0.7	65.6	66.8	1.20	0.7
>12-24	74.7	76.9	2.20**	1.3	72.7	76.0	3.30**	2.0
>24-36	82.5	85.7	3.20**	2.0	81.5	83.8	2.30**	1.4
>36-48	89.6	92.6	3.00**	1.8	87.9	90.3	2.40**	1.5
>48-60	94.8	99.6	4.80**	2.9	94.6	96.9	2.30**	1.4
>60-72	102.1	104.6	2.50**	1.5	100.6	104.1	3.50**	2.1

Level of significance for differences between past and present measurements:

^**^p<0.01

**Fig. 3a.**
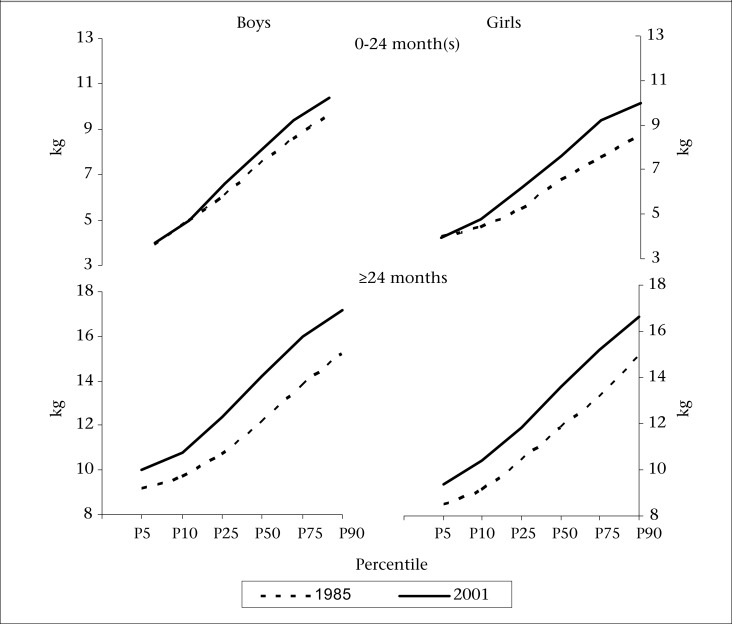
Percentile values for distribution of weight among preschool children in two studies—1985 and 2001

**Fig. 3b.**
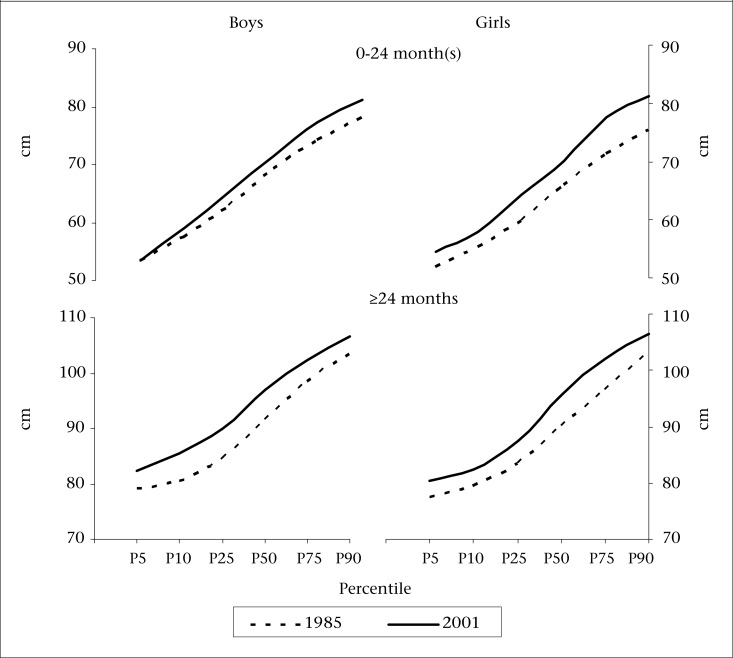
Percentile values for distribution of height among preschool children in two studies—1985 and 2001

The proportions of wasting and stunting based on z-scores observed in the two studies show (Table 4) that the overall prevalence of wasting (weight-for-age z-score <-2) reduced significantly in boys (from 32.1% to 10.3%; p<0.001) as well as in girls (from 19.0% to 9.8%; p<0.001). Maximum reduction in the prevalence of wasting was seen among children beyond infancy. On the other hand, although reduction in the overall prevalence of stunting (height-for-age z-score <-2) was significant, it was smaller among girls (from 54.0% to 39.1%; p<0.001) than boys (from 59.5 to 34.6%; p<0.001) and was maximum among male infants.

## DISCUSSION

Secular trends in growth are known to be predominantly, if not wholly, reflection of changes in living conditions of a population rather than a reflection of any hypothetical shifts in its genetic make-up (11,12). As such, these provide means of judging whether or not the overall development in the given country or region has been able to bring about concomitant changes in health status of its people. Although India has witnessed considerable progress in the post-independence period, undernutrition in preschool children, especially in rural areas, continues to be a persistent problem. We, therefore, examined secular changes in growth of rural preschool children from the villages near Pune city over the period 1985-2001. The observations indicate significant secular changes in height and weight but were more prominent in older children than younger ones, i.e. children below 2 years of age. Further, these changes were smaller in magnitude for girls compared to those observed for boys.

**Fig. 4.**
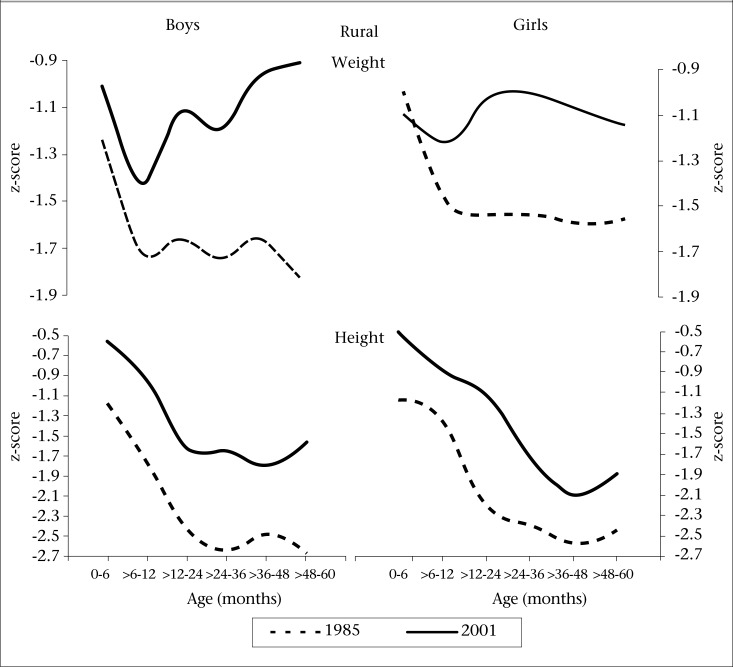
Mean weight-for-age and height-for-age z-scores among preschool children in the two studies—1985 and 2001

Traditionally, secular trend estimates are obtained from repetitive cross-sectional studies. Although our sample-size was smaller compared to such other cross-sectional studies, it had the benefit of reporting data on the same rural community with the same investigators who were present in the past and the present study. It is known (13) that in studies with small sample-size, the accurate reflection of the children for the area is essential and can be effective if examination of secular changes in growth is focused on data from a single region over a long duration. Strength of the study also lies in the similar protocols and almost similar seasons (generally during winter and early summer) of the 1985 and 2001 studies for data collection, which adds to the reliability and accuracy of the reported results.

Evidence of a secular change is often provided by the shift in the measure of central tendency (mean or median) for a distribution of parameter of interest at two time-points. We observed a shift in median weight and height in both the sexes. However, the quantitative estimates for the shift are not attempted as our study group consists of young children wherein yearly rate of growth in each age-group are known to be significantly different. Therefore, the estimates of shift would be sensitive to even smaller differences in the age distributions of children studied at two time-points. Nevertheless, it certainly indicates that some benefits of development have been reflected in the growth of preschool children from rural areas.

**Table 4.** Proportion of stunting and wasting by age and sex in 1985 and 2001 study

Age (months)	Boys					
	Number		Wasting (%)		Stunting (%)	
	1985	2001	1985	2001	1985	2001
0-12	140	69	28.6	17.4	34.3	20.3
>12-24	116	75	34.5	12.0	67.2	42.7
>24-36	95	69	33.7	17.4	75.8	40.6
≥36	276	293	32.2	6.5	63.4	34.5
Total	627	506	32.1	10.3	59.5	34.6
Girls						
0-12	140	54	14.3	18.5	24.3	20.4
>12-24	83	77	24.1	13.0	62.7	32.5
>24-36	87	66	20.7	13.0	65.5	45.5
≥36	227	251	19.4	6.0	64.8	43.4
Total	537	448	19.0	9.8	54.0	39.1

Decadal changes were negligible in infancy for both the sexes and improved only marginally for children in 1-3 year(s) age-group. Nutritional status in early life is predominantly influenced by breastfeeding and weaning practices. As prolonged breastfeeding is characteristic of rural community in India, the data on breastfeeding and weaning practices which influence growth in infancy and early life, were examined. The mean duration of exclusive breastfeeding observed in the present study (6.7±4.4 months) was significantly lower (p<0.01) compared to that reported in our previous study (11.7±7.2 months). Although the age of complete weaning was earlier in the present study (18.4±7.1 months vs 23.0±7.9 months; p<0.01), it was much later than recommended (WHO). It is known that the age at which complementary feeds are introduced is a sensitive time for growth of infants, since breastmilk alone is insufficient to meet full nutritional needs of infants. Weaning at age beyond 6 months has been shown to increase the likelihood of stunting among rural children in India and other developing countries (14). Studies reported for different rural communities in India (15,16) during 1985 and 2001 not only confirm these estimates of breastfeeding duration but also highlight that initiating supplementary foods—adequate in quantity and quality—at appropriate time is still lacking in rural India (17). Thus, the negligible secular changes observed in children below two years of age possibly reflect that there is hardly any improvement with regard to feeding and weaning practices in rural communities in India.

The mean weights and heights observed in 1985 were slightly lower than the 5^th^ percentile of WHO and were comparable with other studies reported for rural children from different states in India (16,18,19). This suggests that growth of preschool children is typically poor in rural India. The mean measurements in 2001 were significantly higher only beyond 3 years of age when compared with respective means observed in 1985. Nevertheless, the growth curve moved only closer to the 5^th^ percentile. The decadal changes in weight and height were considerably large in older children for both the sexes but were lower for girls than boys. The observed positive trends in growth of older children can be attributed to overall improvement in the socioeconomic conditions, which may improve food availability in these rural families. Observed increase in weight was about 10-15% while that in height was around 3-5% during the study period. These observations were similar to those reported among preschool children from Singapore wherein 4.5% increase in height and 11.5% increase in weight were observed (20) during 1972-1987. Danker-Hopfe and Roczen (2) also observed higher secular trends in 6 years old Bremerhavan boys than girls during 1968-1982 but were much smaller (0.48 kg/decade and 0.67 cm/decade) compared to our estimates. This is probably because, in many European countries, secular trend has started much earlier and are coming close to a halt while India is only recently progressing. The comparison of decadal changes reported in different countries/populations, thus, becomes difficult as it is known to vary due to numerous factors such as time (21), age, race (22), region (23), social class (24), and parents’ education (25).

Comparison of percentiles showed that, among older children, the differences were fairly similar across all the percentiles while, in the case of younger children, these were significant only above the 50^th^ percentile. Although comparison of percentiles has been attempted by few researchers, these are hardly used for estimating secular changes. Weighted estimate of the secular change was, therefore, computed using change in percentile values and respective frequencies represented by percentiles as weights.

The maximum negative weight-for-age z-score occurred at around 2 years of age both in the past and the present study, indicating that age at peak prevalence of wasting continues to be the same. Victora *et al*. (26) analyzed data from 54 countries and reported that height-for-age z-scores were the lowest for children from South-East Asia region and that the peak prevalence of stunting was at around 2 years. The fact that malnutrition is more marked at two years of age was also reported two decades back for rural Coimbatore children (18) as well as for rural Tamilnadu children (27).

Vijayraghavan *et al.* (28) have compared dietary intake data within the family collected in 1996-1997 with data from the same villages collected during 1975-1980 and reported that intakes by preschool children remained inadequate, and there was no significant change. Following growth retardation during infancy, childhood infections, especially diarrhoea, is known to be responsible for the peak prevalence of malnutrition between 2 and 3 years of age (29). Esrey (30) estimated that improvements in sanitation were associated with height-for-age z-score increments of 0.06 to 0.62 in children living in rural areas and 0.26 to 0.65 in children living in urban areas, which are similar to the growth effects of dietary intervention. Our observations, therefore, suggest that significant control over childhood illnesses has not been achieved during the last 15 years. The above findings also raise concern about the coverage of young children through existing nationwide nutrition intervention programme (Integrated Child Development Scheme), and a recent critical appraisal of this programme (31), indeed, concludes that the coverage in 0-3 year(s) of age is poor.

In developing world, poor growth in infancy leads to reduced height-for-age or stunting and is known to be concentrated in the first two years of life. Viewing secular trend in height as a reduction in degree of stunting will allow extrapolating from the mechanisms responsible for stunting to those causing secular height changes (7). Reduction in the prevalence of wasting was more marked (around 68% in boys and 48% in girls) than that in the prevalence of stunting (27% in girls and 42% in boys). In fact, it was observed that progression of stunting continues up to the 4^th^ year of age and that stunting remains a major problem in rural preschool children. Comparative data on childhood malnutrition in India and China at three time-points, viz. 1992-1993, 1998-1999, and 2005-2006 also show that the prevalence is much higher in India and that the progress has been slower (32).

Although India witnesses significant materialistic development, the estimates of secular trends are meagre. Considering that nationwide nutrition intervention programmes for preschool children are in operation for more than two decades in India, our findings showing negligible secular changes in children below two years of age suggest that concentrated efforts for creating health and nutritional awareness among rural mothers are required. Further, relatively higher secular changes observed in weight than height of these rural children have two implications. First, this suggests that, although economic conditions have improved (33), the environmental conditions such as lack of hygiene and sanitation continue to remain poor (as evident from government figures given in Table 1). Humphrey (34), reported that prevention of tropical enteropathy, which afflicts almost all children in the developing world, will be crucial to normalize child growth and that this will not be possible without provision of toilets. Second, if these trends continue, excessive weight in rural children experiencing stunting in early life may increase health risks in their adulthood. Therefore, investments in human resource development are necessary for achieving long-term health benefits for Indian population.

## ACKNOWLEDGEMENTS

We are grateful to Director, Agharkar Research Institute, for providing facilities for this research. We would also like to thank the staff involved in this project as well as the young children and their parents for extending their necessary cooperation.
